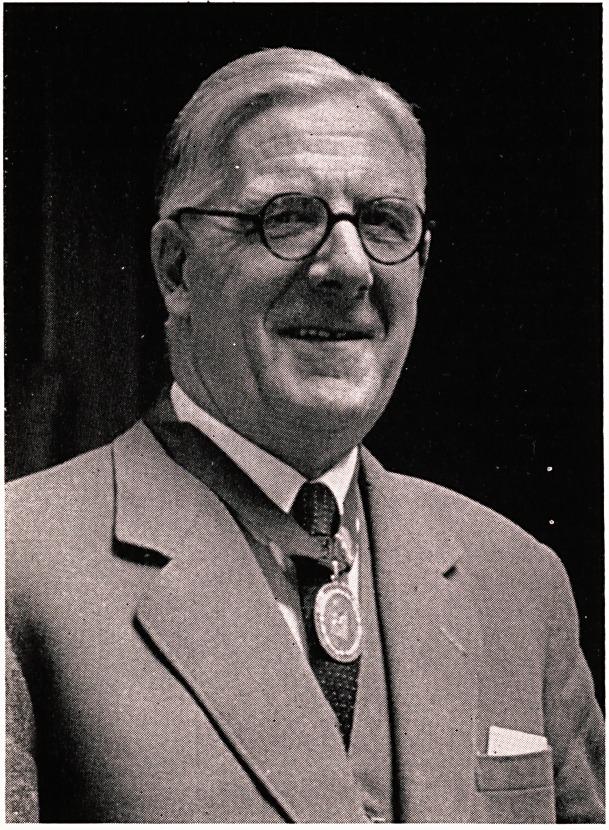# Albert Victor Neale

**Published:** 1970-10

**Authors:** 


### Obituary

### ALBERT VICTOR NEALE

M.D., F.R.C.P., D.P.H.

Emeritus Professor A. V. Neale, who was Professor of Child Health in the University of Bristol from 1947 to 1965 died suddenly at home in Bristol on February 1st, 1970, aged 70.

Albert Victor Neale was born in Birmingham, the seventh in a family of ten children. Despite a lack of the usual educational opportunity his tremendous capacity for hard work and high intellectual calibre enabled him to enter Birmingham University as a Queen's Scholar. He had a brilliant undergraduate career, qualifying M.B., Ch.B., in 1926. Whilst holding resident appointments he gained M.R.C.P. London, D.P.H., and M.D. with honours. Sir Leonard Parsons, who became his wise counsellor and friend, directed his interest towards paediatrics and this was strengthened whilst holding a Rockefeller Fellowship to U.S.A. when he participated in the pioneer research on gastroenterology and fluid balance in children with Gamble at Harvard University.

On his return from U.S.A. he was appointed pathologist to Birmingham Children's Hospital where he quickly established himself as a popular teacher and became very knowledgeable on medico-legal problems. Subsequent appointments as Honorary Physician in Birmingham to the General Hospital and the Children's Hospital enabled him to practise as a general Consultant Physician for adults and children with a great reputation for his kindness to patients. He was elected F.R.C.P. in 1937. A dynamic personality, he was loved by his colleagues who missed him greatly when he left Birmingham in 1947 to become Professor of Child Health in Bristol.

With characteristic energy Victor Neale adapted to his new rôle. Despite the lack of a physical department throughout his whole occupancy of the Chair, with single-minded determination he established Child Health as an academic discipline in Bristol University in a way which has been admired and envied nationally and overseas. His inspired and provocative teaching and versatility in the preparation of curricula were greatly appreciated by undergraduates, and general practitioners; many senior teachers of paediatrics in developing countries remember with affection and gratitude their postgraduate training in Bristol. He was a popular Dean of the Faculty of Medicine from 1956-1961 and often students and young members of staff sought his advice. He examined for the Royal College of Physicians of London and several Universities.

As an astute and extremely well-informed clinician his interests were catholic, but with special emphasis on social paediatrics as shown in his Long Fox Lecture and his Heath Clark Lectures in London University in 1961. He did much to inspire and help the pioneer work of others in neonatology, psychosomatic disorders and cerebral palsy particularly. At the bedside or in the clinic his fatherly tenderness endeared him to the children, who often found a sixpence in their palms when he left them. No parental anxiety was too trivial to be dismissed without a word of kindly advice; such occasions were always turned to good effect for teaching students by example as well as precept.

Whilst he was always certain that the hub of his activity should be firmly embedded in the Bristol Royal Hospital for Sick Chilren, the spokes of the wheel of his life radiated in every direction so that nothing that impinged on knowledge of children, their needs in health and disease and the effects of environment on them escaped his influence. To ensure this global view of child health he was frequently to be found "perambulating the periphery" in such diverse activities as talks to parent-teacher groups, the Chairmanship of the Bristol Marriage Guidance Council, and editor of the report of a sub-committee on Geriatrics. He had a gift for stimulating co-operative activity among people with different interests so that the recurrent theme of "interdepartmental interdigitation" became well known among his University and Hospital colleagues, particularly in the Departments of Obstetrics and Public Health. His department was a centre to which all those concerned with Child Health in the Bristol clinical area and in the South Western Region would automatically gravitate for mutual assistance, and friendship. The South West Paediatric Club is an enduring example of the success of his efforts to create a real family of paediatricians for the Region.

He was much sought after as a shrewd committee man and administrator whose penetrating mind went swiftly to the core of any problem, though the breadth and length of his vision was often ahead of his time. As President of the British Paediatric Association in 1961 he stimulated new ideas concerning the role of Paediatricians in Britain. He served on the South Western Regional Hospital Board and on the Board of Governors of United Bristol Hospitals. As a member of Southmead Hospital Management Committee from 1948 he made a great contribution to the development of that hospital, particularly since its recognition for undergraduate clinical teaching, and at the time of his death he was Acting-Chairman. He undertook tours of the West Indies and Pacific Islands as advisor to the Government.

After his retirement in 1965 he continued as energetically as ever to advise on the establishment of a department of Child Health in Newfoundland, and was subsequently W.H.O. Professor in Khartoum for two years.

A sensitive man, Victor Neale spurned ostentation and luxury but had a tremendous sense of purpose in life; he made decisions boldly and had a gift for persuading others to help him. As long as there was a job to be done he could not relax. His understanding and humour endeared him to all and there was invariably a cheery word and many kindly acts for even the humblest members of the community. He had little time for hobbies, but enjoyed music and the theatre, and tackled his garden with his usual efficiency.

He married Phyllis Ball, one of his students in Birmingham and also a Queen's Scholar. They enjoyed an ideally happy home life with their three daughters, two of whom are social workers, and one a district nurse. His greatest pleasure and relaxation was to sit by the fire with a pile of books, for he was a voracious reader, in the company of his family. To them we offer our heartfelt sympathy.

## Figures and Tables

**Figure f1:**